# Graphic Medicine (website)

**DOI:** 10.29173/jchla29926

**Published:** 2026-08-03

**Authors:** Mackenzie Hilton

**Affiliations:** The Centre for Addiction and Mental Health (CAMH), Toronto, ON, Canada

**Product:** Graphic Medicine (website)

**URL:** https://www.graphicmedicine.org/

## Purpose

The purpose of this review is to highlight GraphicMedicine.org and elucidate how this website serves as a valuable advisory tool for health science libraries.

## Product description

GraphicMedicine.org is a “website that explores the interaction between the medium of comics and the discourse of healthcare” [1]. Graphic medicine constitutes a subgenre of graphic novels that utilize the graphic format of comic books. These graphic novels are visually appealing, accessible, and cover a wide range of topics including mental health, grief and loss, public health, disease, 2SLGBTQIA+ health, Indigenous health, and aging, to name a few. This makes graphic medicine a welcomed addition to any health sciences library. GraphicMedicine.org provides lists of new and older graphic medicine novels with corresponding reviews, metadata, and purchase information. GraphicMedicine.org also functions as a community hub by hosting annual conferences, producing podcast episodes, and circulating newsletters.

## Intended users

GraphicMedicine.org is intended for anyone interested in graphic medicine novels and comics generally. All features are free to use, meaning anyone can view book lists and reviews, and even submit reviews themselves. This makes GraphicMedicine.org a highly accessible advisory tool.

## Special features

GraphicMedicine.org offers many features that users, especially health science library staff, will find helpful.

### Reviews

**Fig. 1:**
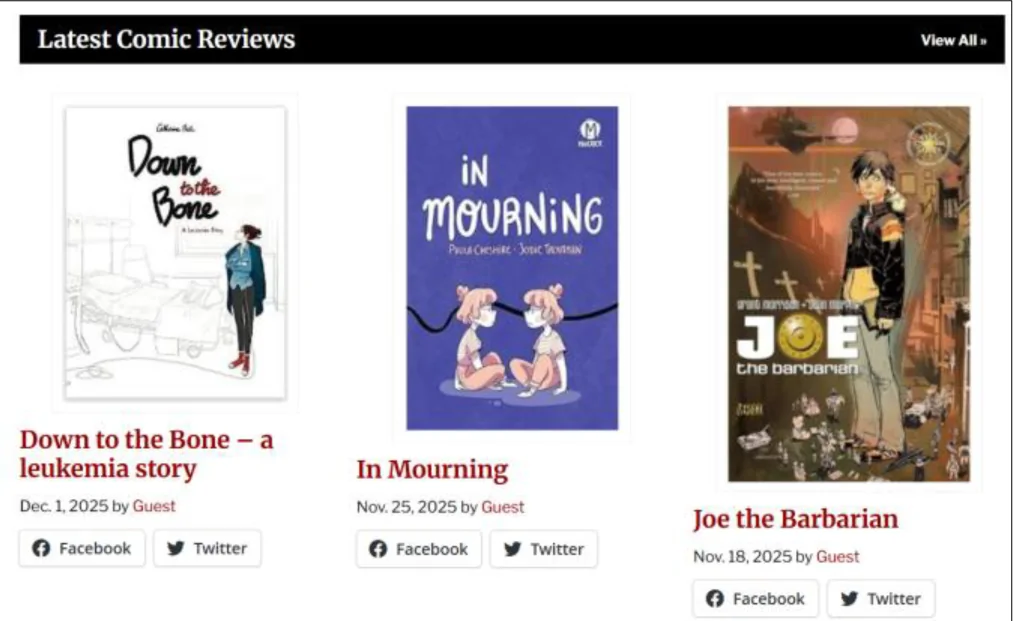
Latest comic reviews

GraphicMedicine.org organizes reviews into the following categories: Editor’s Picks, Graphic Novels, Comic Books (Figure 1), Educational, Manga, Bandes Dessinèes (French comics), Web Comics, and Picture Books. These categories provide quick access to specific formats. The Editor’s Picks category, for example, provides users with a good starting point for selecting graphic novels to read or add to a collection. There is also a list of “unreviewed graphic novels” which may encourage users to write and submit a review of their own.

The reviews are extensive and detailed, containing a synopsis of the graphic novel as well as information regarding the book format, page count, publisher information, and where it can be purchased.

### Annotated bibliographies

“Essential Graphic Medicine: An Annotated Bibliography” consists of 30 bibliographic entries of “must-have comics for a graphic medicine collection” [2]. The list is intended to help libraries start a graphic medicine collection. The titles included in this list are the result of a survey with over 90 participants to minimize biases.

### Syllabus repository

**Fig. 2:**
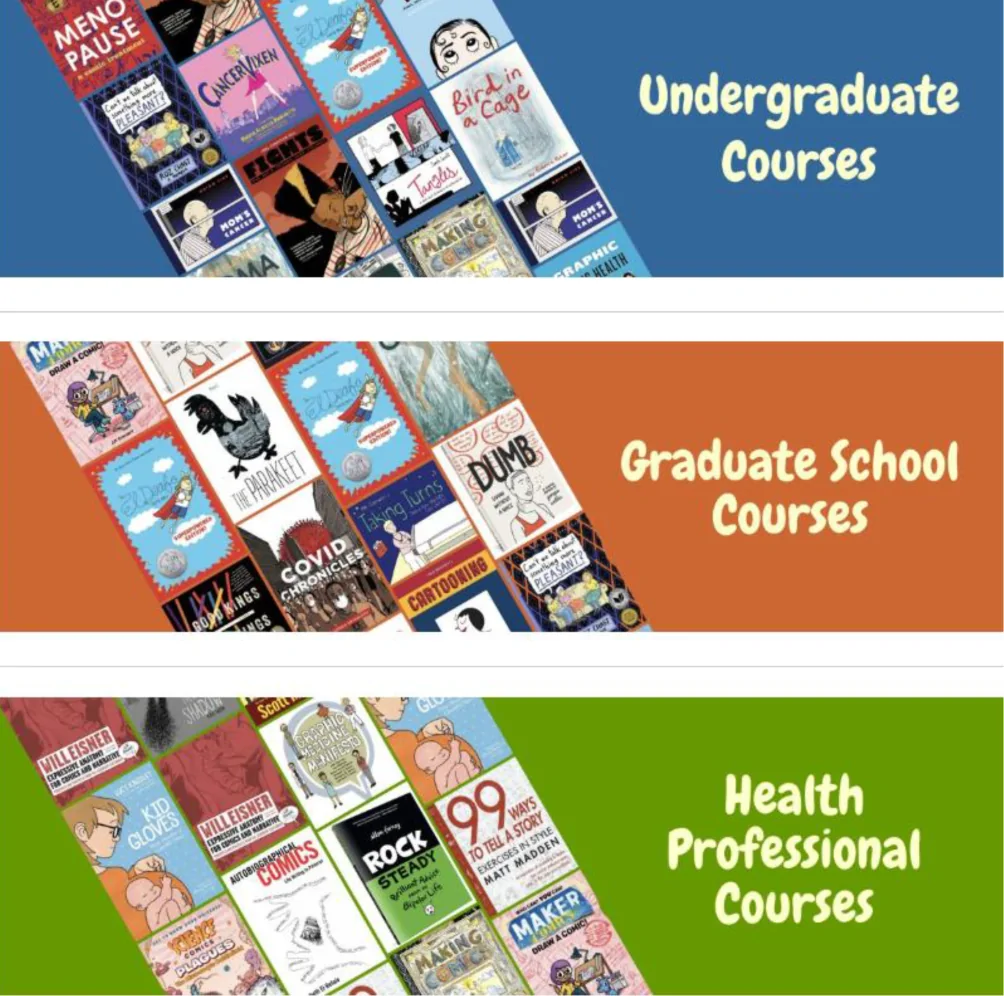
The syllabus repository’s three categories

As the popularity of graphic medicine novels grows, more undergraduate, graduate, and professional programs are offering courses related to comics and medicine [3]. GraphicMedicine.org includes a repository of syllabi created and used by instructors of these courses. These resources are collected and shared by the Graphic Medicine International Collective (GMIC). Syllabi are organized into three categories: Undergraduate Courses, Graduate Courses. and Health Professional Courses (Figure 2). Each syllabus lists the title of the course, the course instructor, the institution, and the year the course was taught. This provides a quick way to determine the themes of the syllabi (e.g. Health Humanities, COVID, Disability) and to ensure that the syllabi are up-to-date and relevant.

### Liaison Program

**Fig. 3:**
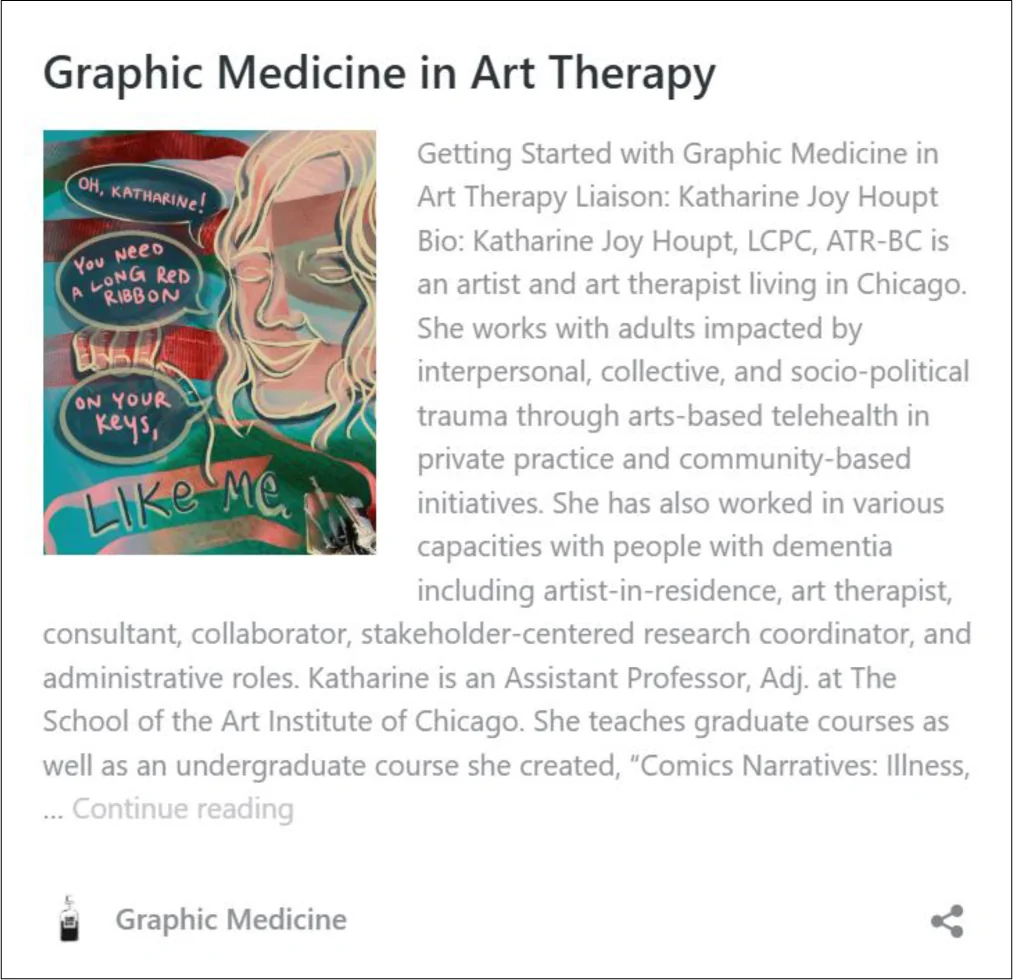
Liaison program for Graphic Medicine in Art Therapy

The Liaison Program consists of a community of graphic medicine specialists who each specialize in a different area of graphic medicine. Liaisons serve as a point of contact for 11 different fields, including Graphic Medicine in Art Therapy (Figure 3), Graphic Medicine in Bioethics, and Graphic Medicine in Nursing. Users interested in any of these fields can contact the liaison to learn more about how graphic medicine intersects with the specialization. Each liaison page includes a bio of the liaison, their contact information, a description of their field, and resources on how to incorporate graphic medicine into the specific field of practice.

### Graphic Medicine newsletter

Users can sign up for a weekly e-newsletter. Newsletters include information about upcoming events, ways for users to get involved, and deadlines for awards, conference submissions, and other important news.

### Podcasts

**Fig. 4:**
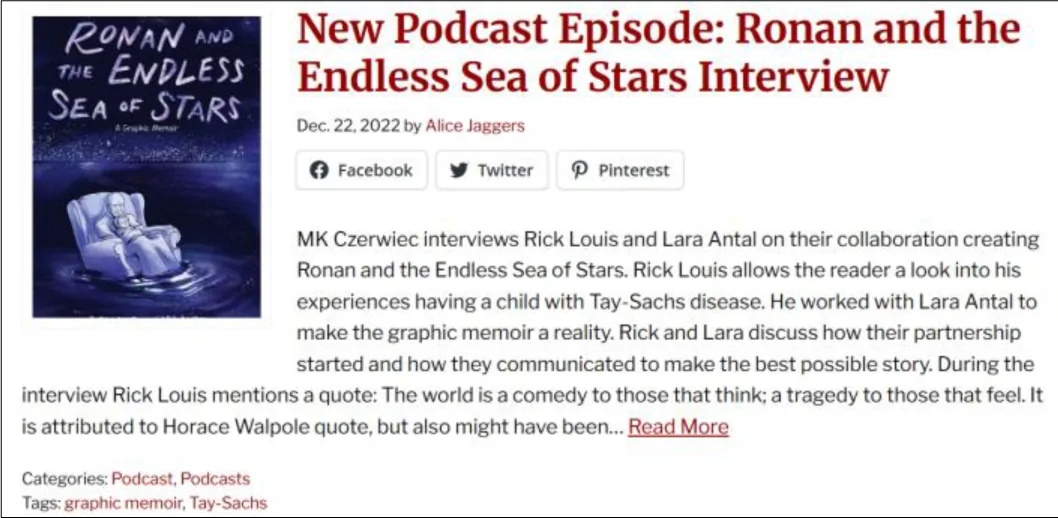
Podcast episode featuring an interview

Podcast episodes range from one to two hours in length, and cover a variety of topics, including book discussions, health conditions (like diabetes), and specialized topics, such as creating a graphic medicine course. Episodes include a description of the content, as well as a list of the speakers (Figure 4).

### Drawing Together

**Fig. 5:**
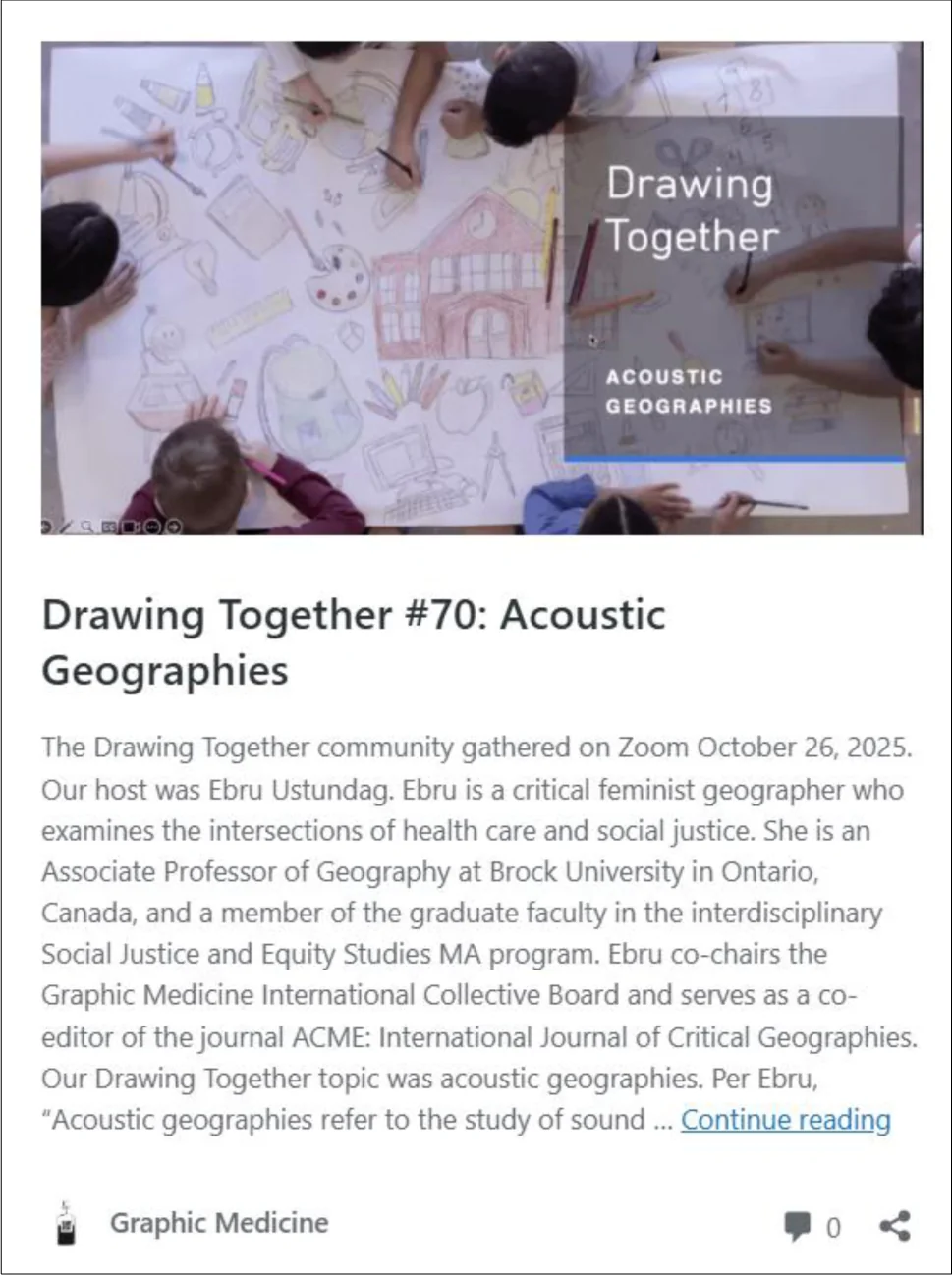
Drawing Together Session from November 2025

Drawing Together (Figure 5) is a free, virtual meeting that takes place on Zoom on the last Sunday of every month at 1300 h EST. Users can sign up for an email list to receive updates. Each month, a new topic and group facilitator is assigned, and users meet on Zoom to draw, support one another, and share their thoughts about the topic at hand [4].

### Other special features

In addition to the features listed above, GraphicMedicine.org includes graphic medicine exhibits, annual conferences, resources on publishing graphic novels, video archives, awards, and scholarships. GraphicMedicine.org truly is a treasure trove of resources!

## Compatibility

GraphicMedicine.org can be accessed on any internet browser, or on any device with an internet connection. This includes desktop computers and phones.

## Usability

Clearly labelled tabs and categories make accessing information quick and easy. GraphicMedicine.org also includes a search bar that can be used to search for specific titles, topics, and authors.

## Strengths and weaknesses

### Strengths

- Easy to navigate
- Freely accessible
- Book lists and editor recommendations provide good starting points for building a graphic medicine collection
- Reviews state advisory warnings, such as obscene or sexual content
- Reviews are published regularly, covering a variety of old and new titles
- Authors and illustrators of graphic novels are credited and have their personal website and information included in reviews
- Each review includes images of the book, providing a snapshot of its illustrations
- Website alternatives are provided for Spanish, Japanese, and Italian resources
- Users can contribute to GraphicMedicine.org by submitting reviews of their own, and attending the annual conference

### Weaknesses

- Some books lists and resources, such as the annotated bibliography of “must-have titles,” are not updated regularly

## Cost/value

GraphicMedicine.org and all its features, including its reviews, booklists, podcast episodes, newsletters, and conferences are free to access.

## Conclusion

GraphicMedicine.org provides users with a quick, efficient, and free way to view popular graphic medicine titles. GraphicMedicine.org can and should be consulted when developing a graphic medicine collection, or simply selecting graphic medicine titles to read. GraphicMedicine.org can also be used as a resource for developing courses. The website serves as a valuable advisory tool, and also connects users with similar interests through conferences, drawing together sessions, and liaison programs.
